# Rapid Acquisition of High-Pixel Fluorescence Lifetime Images of Living Cells via Image Reconstruction Based on Edge-Preserving Interpolation

**DOI:** 10.3390/bios15010043

**Published:** 2025-01-13

**Authors:** Yinru Zhu, Yong Guo, Xinwei Gao, Qinglin Chen, Yingying Chen, Ruijie Xiang, Baichang Lin, Luwei Wang, Yuan Lu, Wei Yan

**Affiliations:** 1 State Key Laboratory of Radio Frequency Heterogeneous Integration (Shenzhen University), Key Laboratory of Optoelectronic Devices and Systems of Ministry of Education and Guangdong Province, College of Physics and Optoelectronic Engineering, Shenzhen University, Shenzhen 518060, China; 2150453014@email.szu.edu.cn (Y.Z.); 1800284004@email.szu.edu.cn (Y.G.); 2250453008@email.szu.edu.cn (X.G.); chen13d@gmail.com (Q.C.); 2310452021@email.szu.edu.cn (Y.C.); 2200453039@email.szu.edu.cn (R.X.); linbaichang2022@email.szu.edu.cn (B.L.); wanglowell@szu.edu.cn (L.W.); 2 The 6th Affiliated Hospital of Shenzhen University, Huazhong University of Science and Technology Union Shenzhen Hospital, Shenzhen 518060, China

**Keywords:** fluorescence lifetime imaging, image reconstruction, live-cell imaging, high-pixel image, edge-preserving interpolation method

## Abstract

Fluorescence lifetime imaging (FLIM) has established itself as a pivotal tool for investigating biological processes within living cells. However, the extensive imaging duration necessary to accumulate sufficient photons for accurate fluorescence lifetime calculations poses a significant obstacle to achieving high-resolution monitoring of cellular dynamics. In this study, we introduce an image reconstruction method based on the edge-preserving interpolation method (EPIM), which transforms rapidly acquired low-resolution FLIM data into high-pixel images, thereby eliminating the need for extended acquisition times. Specifically, we decouple the grayscale image and the fluorescence lifetime matrix and perform an individual interpolation on each. Following the interpolation of the intensity image, we apply wavelet transformation and adjust the wavelet coefficients according to the image gradients. After the inverse transformation, the original image is obtained and subjected to noise reduction to complete the image reconstruction process. Subsequently, each pixel is pseudo-color-coded based on its intensity and lifetime, preserving both structural and temporal information. We evaluated the performance of the bicubic interpolation method and our image reconstruction approach on fluorescence microspheres and fixed-cell samples, demonstrating their effectiveness in enhancing the quality of lifetime images. By applying these techniques to live-cell imaging, we can successfully obtain high-pixel FLIM images at shortened intervals, facilitating the capture of rapid cellular events.

## 1. Introduction

The long acquisition time required for fluorescence lifetime images has always been an important issue demanding urgent resolution [1]. The fluorescence lifetime, which represents the duration for a fluorescent molecule to relax from its excited state back to the ground state, is a statistical outcome derived from measurements across numerous molecules and thus requires an ample count of photons for calculation; this typically requires the superposition of multiple image frames [2]. Most acquisitions of lifetime images are based on continuous scanning via a laser scanning confocal microscope (LSCM) equipped with a time-correlated single-photon counting (TCSPC) module [3]. This approach, however, poses two salient challenges. First, continuous laser scanning can induce photo-toxicity and dye bleaching. Second, rapid cellular activity leads to subtle variations between frames, resulting in blurred cell structure edges in the final images.

To address these issues, strategies include employing dyes that are less prone to bleaching and exhibit a brighter fluorescence [4,5,6,7], coupled with faster imaging modalities which reduce the scanning time [8,9,10,11,12]. Nevertheless, the quest for higher-resolution imaging readily undermines efforts aimed at shortening scan times, as even a modest twofold increase in scanning pixels can exponentially increase the scanning time by more than the cube of this factor—with twofold increments from the *x*-axis and another twofold from the *y*-axis. Practically, compromises are often made by reducing the maximum photon count to curtail the acquisition time, which may reduce the brightness of the image and introduce large errors in lifetime fitting.

The interpolation of low-resolution (LR) images is imperative to acquire high-pixel images of specific regions within a short acquisition time. Image reconstruction methods that utilize deep learning have been reported. Dong et al. reported the interpolation and evaluation of fluorescence lifetime images based on semi-synthetic data [13]. However, actual intracellular conditions are far more complex, and there remains an unresolved issue regarding the degree of deviation between true and predicted values. Currently, post-processing in fluorescence lifetime imaging focuses on the rapid fitting of fluorescence lifetimes [14] or the design and application of non-fitting methods such as phasor tools [15,16,17,18]. There remains a lack of reasonable interpolation methods for fluorescence lifetime images that can quickly obtain high-pixel images from low-resolution images while being closer to the true values.

In this article, we employ the traditional bicubic interpolation method to interpolate both the fluorescence intensity and fluorescence lifetime of fluorescence microspheres and fixed-cell samples separately. Subsequently, we adopt an image reconstruction approach based on the edge-preserving interpolation method (EPIM) [19] to obtain high-pixel lifetime images. These images are then compared with those obtained using bicubic interpolation. Finally, we validate the effectiveness of our method in the continuous imaging of living cells.

## 2. Materials and Methods

### 2.1. Bicubic Interpolation Algorithm

Fluorescence lifetime images typically contain intensity information derived from the superposition of multiple intensity frames and lifetime information obtained through a fitting process, as described previously [20,21]. MATLAB (version, R2018a) is utilized to interpolate the extracted fluorescence intensity and lifetime information separately.

The bicubic interpolation algorithm uses the values of 16 known pixels within a 4 × 4 neighborhood centered on the interpolation point as references and then performs third-order interpolation separately in both the horizontal and vertical directions. The calculation formula is as follows [22,23,24]:

(1) $$f\left(i+s,j+t\right)=ABC$$

(2) $$A=\left[R\left(s+1\right)R\left(s+0\right)R\left(s-1\right)R\left(s-2\right)\right]$$

(3) $$B=\left[\begin{array}{cc} \begin{array}{cc} f\left(i-1,j-1\right) & f\left(i-1,j-0\right)\\ f\left(i+0,j-1\right) & f\left(i+0,j-0\right) \end{array} & \begin{array}{cc} f\left(i-1,j+1\right) & f\left(i-1,j+2\right)\\ f\left(i+0,j+1\right) & f\left(i+0,j+2\right) \end{array}\\ \begin{array}{cc} f\left(i+1,j-1\right) & f\left(i+1,j-0\right)\\ f\left(i+2,j-1\right) & f\left(i+2,j-0\right) \end{array} & \begin{array}{cc} f\left(i+1,j+1\right) & f\left(i+1,j+2\right)\\ f\left(i+2,j+1\right) & f\left(i+2,j+2\right) \end{array} \end{array}\right]$$

(4) $$C={\left[R\left(t+1\right)R\left(t+0\right)R\left(t-1\right)R\left(t-2\right)\right]}^{T}$$

(5) $$R\left(x\right)=\left\{\begin{array}{c} \;\;\;\;\;\;1-2{\left|x\right|}^{2}+{\left|x\right|}^{3}\;\;\;\;\;\;\;\;\;\;0\leq \left|x\right|<1\\ 4-8\left|x\right|+5{\left|x\right|}^{2}-{\left|x\right|}^{3}\;\;\;0\leq \left|x\right|<1\\ 0\;\;\;\;\;\;\;\;\;\;\;\;\;\;\;\;\;\;\;\;\;\;\;\;\;\;\;\;\left|x\right|\geq 2 \end{array}\right.$$

Upon acquiring the interpolated results for both grayscale values and lifetimes, the corresponding RGB values in the pseudo-color encoding table are identified based on the grayscale and lifetime values of each individual pixel. These identified RGB values are subsequently utilized to construct a novel pseudo-color interpolated image.

### 2.2. Edge-Preserving Interpolation Method (EPIM)

**Figure 1:**
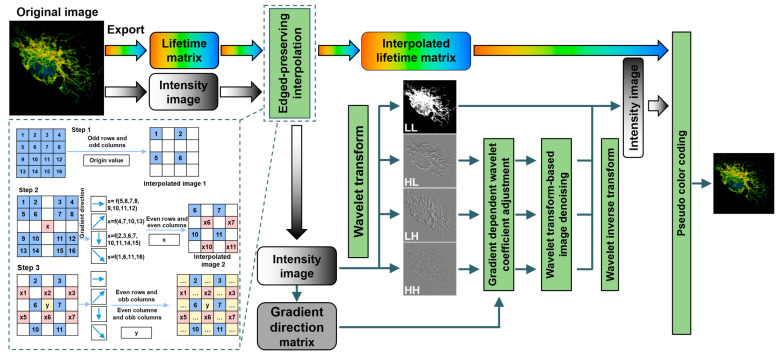
Workflow of image reconstruction based on edge-preserving interpolation. The green boxes represent data processing operations. The colored, black-and-white, and gray rounded rectangles denote the lifetime information, intensity information, and gradient orientation information of the fluorescence lifetime images, respectively. The dashed blue box presents the workflow of the adopted edge-preserving interpolation method. The blue squares contain the data from the original low-resolution image, which are directly assigned to the odd rows and columns of the high-resolution image. The pink squares contain the data from the first step of interpolation, their calculation involving the surrounding 4 × 4 pixels of the original image, and these data are interpolated to the even rows and columns of the high-pixel image. The yellow squares contain data from the second step of interpolation, calculated based on a 5 × 5 neighborhood in the high-pixel image, and these data are distributed on the odd rows and even columns or even rows and odd columns of the high-resolution image.

The workflow of the EPIM is illustrated within the dashed box in Figure 1. After exporting intensity and lifetime information from the fluorescence lifetime image, we performed an interpolation on both intensity and lifetime separately. We then took the example of expanding the image to twice its original size. In Step 1, to preserve more information from the original data, the value of the pixels in the original image was directly assigned to the pixels located on odd rows and odd columns in the interpolated image. In Step 2, the gradient direction of each pixel was calculated to determine the interpolated values for the pixels on even rows and even columns. The magnitude of change in various directions was calculated as follows:

Firstly, the 16 known pixels within a 4 × 4 neighborhood centered on the interpolation point were transformed into a 16 × 1 matrix according to their respective directions. For example, the corresponding 16 × 1 matrix in the horizontal direction was constructed as follows:

(6) $$\left[\begin{array}{ccc} f\left(i-1,j-1\right) & \ldots & \begin{array}{ccc} f\left(i+4,j-1\right) & f\left(i+4,j-0\right) & \begin{array}{ccc} \ldots & f\left(i-1,j-0\right) & \begin{array}{cc} f\left(i-1,j-1\right) & \ldots \end{array} \end{array} \end{array} \end{array}\right]$$

Subsequently, the absolute differences between each adjacent pair of elements were calculated, and their sum was determined.

After computing the magnitudes, the direction with the maximum magnitude was taken as the gradient direction for the pixel. Based on this gradient direction, pixels were selected for calculating the interpolated value using bicubic interpolation. For instance, when the gradient direction was 45°, pixels 1, 6, 11, and 16 were chosen for weighting calculation with weights of (−1/16, 9/16, 9/16, and −1/16). In the case of a horizontal direction, eight pixels along the horizontal were considered, with weights of (−1/16, −1/16, 9/16, 9/16, 9/16, 9/16, −1/16, and −1/16) applied. Then, the calculated value were interpolated to the pixels located on even rows and even columns of the high-pixel image.

In Step 3, the interpolated values for pixels situated on odd rows and even columns, as well as those on odd columns and even rows, were determined. A 5 × 5 window of pixels encompassing the target pixel to be interpolated was identified within the partially interpolated image. Employing a methodology similar to Step 2, the gradient direction was computed based on the already-interpolated pixels. Then, the interpolation value for the target pixel was subsequently calculated and assigned. With this, edge-preserving image interpolation was deemed complete. It is noteworthy that, during the interpolation of the lifetime, pixels with excessively low fluorescence intensities, unsuitable for fluorescence lifetime fitting, were excluded from all computations within the algorithm to avoid potential errors.

### 2.3. Image Reconstruction

The principles and application of wavelet transform have been extensively reported previously [25]. Herein, we applied this transform to the intensity image after edge-preserving interpolation to obtain horizontal high-frequency details, vertical high-frequency details, and diagonal high-frequency details. In the preceding step of EPIM, the gradient direction for each pixel had been obtained. By adjusting the wavelet transform coefficients according to the gradient values and then performing a wavelet inverse transform, an edge-enhanced fluorescence -intensity image was acquired. Additionally, a reconstructed high-pixel image was obtained after denoising.

### 2.4. Fluorescence Lifetime Imaging System Setup

We employed a commercial fluorescence lifetime imaging system (DCS-120 Confocal FLIM System, Becker & Hickl Company, Berlin, Germany) for imaging. A mode-locked supercontinuum laser (SC-PRO-M, Wuhan Yangtze Soton Laser Co., Ltd., Wuhan, China) was utilized as the excitation source, featuring a pulse width of 6 picoseconds and a repetition rate of 80 MHz. This laser was coupled with an acousto-optic tunable filter (AOTF) to facilitate the selection of specific excitation wavelengths, enabling versatile applications across various samples.

For the fluorescent microspheres, fixed-cell samples, and living-cell samples, tailored excitation wavelengths of 570 nm, 633 nm, and 633 nm, respectively, were employed. To effectively isolate the signal from the reflected laser, appropriate bandpass or long-pass filters (620/60, 670/30, and 670/30, respectively) were implemented.

Optical resolution was maximized through the use of a high-performance 100× oil immersion objective lens (NA 1.40, Nikon, Tokyo, Japan). The resulting fluorescence signals were captured by a sensitive hybrid detector (HPM-100, Becker & Hickl GmbH, Berlin, Germany), ensuring accurate and reliable measurements. We collected fluorescence lifetime images with pixels of 256 × 256 and 512 × 512. The 256 × 256 pixel image output by the SPCImage software (version 7.1) were used as the low-resolution images when the amplification factor was set to 2. The 128 × 128 images obtained by downsampling were used as low-resolution images when the amplification factor was set to 4. Their grayscale values and lifetime data were interpolated to obtain a 512 × 512 pixel image. The 512 × 512 pixel image served as the ground truth (GT) for the high-pixel image.

The entire fluorescence lifetime imaging process was seamlessly orchestrated by the SPCM64 software (version 9.76, Becker & Hickl, GmbH, Berlin, Germany), providing intuitive control over data acquisition and data processing.

### 2.5. Sample Preparation

The fluorescent microsphere sample was a mounted sample from the Thermo Fisher Scientific Company. We chose fluorescent microspheres with a size of 4.0 μm from position 1 of a TetraSpeck™ Fluorescent Microspheres Size Kit (Thermo Fisher Scientific Inc., Waltham, MA, USA) for imaging.

The fixed-cell sample with mitochondria specifically labeled was purchased from the Standard Imaging Company. BSC-1 cells were cultured in Dulbecco’s Modified Eagle’s Medium (DMEM) supplemented with 10% fetal bovine serum (FBS). To prevent bacterial contamination, 100 μg/mL penicillin and streptomycin was added in the DMEM. Cells were grown under standard cell culture conditions (5% CO_2_, humidified atmosphere at 37 °C). BSC-1 cells were plated on a #1.5 glass-bottom dish for 48 h before sample preparation. On the day of sample preparation, the cells were fixed with 37 °C pre-warmed fixation buffer for 10 min, containing 4% paraformaldehyde and 0.1% glutaraldehyde in PBS. Then, the samples were washed three times with PBS. For quenching the background fluorescence, we incubated the cells with 2 mL 0.1% NaBH4 solution in PBS for 7 min. The samples were washed three times with 2 mL PBS and then incubated for 30 min in PBS containing 5% BSA and 0.5% Triton X-100 (SunBloss, South Orange, NJ, USA, HXKx02) at 37 °C. All antibodies were diluted in the 5% BSA + 0.5% triton solution. Next, we incubated the sample for 40 min with the appropriate dilution of primary antibodies—beta-tubulin (DSHB-E7), Tom20 (abclonal, A19403), and LAMP1(abclonal, A22482)—at 25 °C. After the incubation of primary antibodies, the cells were washed for 5 min with 2 mL PBS, three times. The secondary antibodies, specifically abberior STAR RED, goat anti-mouse IgG (Abberior, STRED-1001-500UG) and abberior STAR RED, goat anti-rabbit IgG (STRED-1002-500UG), were incubated with the appropriate dilutions for 60 minutes at a temperature of 25°C. During this incubation, the samples were kept in the dark to prevent photobleaching. Following three washes with PBS, the cells underwent fixation with a post-fixation buffer for a duration of 10 minutes. The fixed-cell sample utilized for testing the nuclear pore complex (NPC) was a standard CELLS 4C sample that had been obtained from Abberior GmbH. The channel corresponding to NPC-STAR RED was selected for imaging.

Living HeLa cells cultured in Dulbecco’s Modified Eagle’s Medium (DMEM) containing 10% fetal bovine serum (FBS) and 1% penicillin/streptomycin were stained with 10 μL of 75 nM Mito-Tracker Deep Red dye. Following incubation for 10 min at 37 °C in a 5% CO_2_ environment, the stained cells were washed three times with PBS, replenished with the same DMEM, and further incubated for 10 min to maintain cell viability and reduce stress responses for better living-cell imaging.

## 3. Results and Discussion

### 3.1. Fluorescent Microsphere Samples

**Figure 2:**
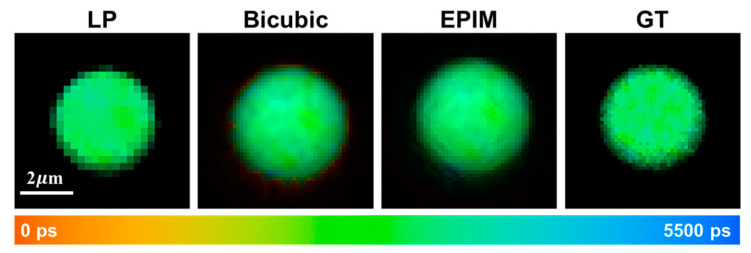
Fluorescence lifetime images of microsphere captured at a low resolution (LP) of 256 × 256 pix-els, processed using bicubic interpolation, reconstructed through EPIM, and acquired at a high resolution serving as the ground truth (GT), respectively.

Initially, we validated our methodology on individual fluorescent microspheres. As illustrated in Figure 2, the fluorescence lifetimes among individual pixels within the fluorescent microspheres exhibited a high degree of uniformity. In regions outside the microspheres, due to the insufficient accumulation of photons, the calculation of fluorescence lifetime values was infeasible. The direct magnification of low-resolution images revealed pronounced variations in brightness at the microspheres’ edges, accompanied by jagged edge artifacts. However, the bicubic interpolation algorithm addressed this issue effectively, resulting in smoother edge transitions in the intensity images. Nevertheless, due to the specific nature of bicubic interpolation, pixels surrounding the microspheres that lacked calculated fluorescence lifetime values were inadvertently included in the computation, leading to significant discrepancies in the fluorescence lifetime between the microspheres’ edges and their center. These edge pixels, despite having non-zero intensities after computation, exhibited undesirable abrupt changes in pseudo-color images and did not represent the actual lifetime values of the component at the edge locations. Therefore, pixels with insufficient photons for lifetime calculation in the original image periphery must be excluded from the interpolation algorithm.

To address this, we employed a reconstruction algorithm based on edge-preserving interpolation. Prior to computing the fluorescence lifetime at the interpolation points, pixels with low photon counts in the vicinity were excluded. The absence of these points during computation was mitigated by assigning higher weights to other pixels. This approach successfully mitigated the abrupt changes in fluorescence lifetime at image edges introduced by the bicubic interpolation algorithm. The intensity images, after undergoing various processing steps, recovered information lost from edge pixels due to low-resolution acquisition, albeit resulting in less-smooth edges. When compared with GT images, it was evident that the bicubic interpolation algorithm overly smoothed the edges, whereas the images obtained using the EPIM more accurately simulated the complex gradient variations at the image edges.

### 3.2. Fixed-Cell Samples

**Figure 3:**
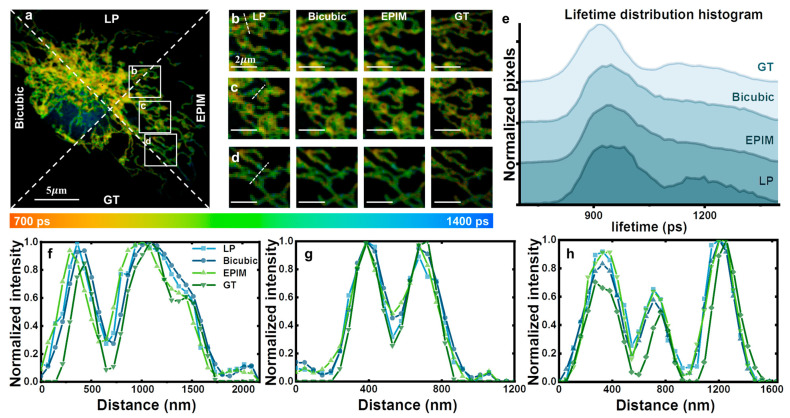
(**a**) Fluorescence lifetime image comparison of the LP, bicubic-interpolated, EPIM reconstruction, and GT images, divided by dotted lines. The amplification factor was set to 2. (**b**–**d**) ROIs of (**a**). (**e**) Fluorescence lifetime distribution histogram of (**a**). (**f**–**h**) Spatially resolved analysis at the white dashed line in (**b**), (**c**), and (**e**), respectively.

Next, we conducted an investigation into image reconstruction for fixed cellular samples. The system configuration setup remained unchanged, except for the excitation wavelength being switched to 633 nm and the use of a 670/30 bandpass filter. Detailed information on sample preparation is provided in the Supplementary Materials. Since the dye exclusively targeted the mitochondria, the fluorescence lifetime was predominantly in the range from 700 ps to 1400 ps (Figure 3). The background fluorescence, mainly centered around the nucleus, exhibited a lifetime substantially exceeding 1400 picoseconds. We chose the LP image, the bicubic-interpolated image, the EPIM image, and the GT for comparison (Figure 3a). Regions of interest (ROIs) were selected within the samples. The low-resolution images exhibited notable jagged edges in some areas, shown in Figure 3b–d, which was an artifact of direct image magnification. The bicubic interpolation algorithm resulted in a smooth transition in both the lifetime values and the grayscale intensity of the image pixels, causing the mitochondrial edges to appear relatively blurred. Due to the continuity of lifetimes within this image, significant interpolation errors, akin to those observed in fluorescent microspheres, were absent. In the GT images, the distribution of fluorescence lifetime and intensity across different points did not follow a simple, completely smooth gradient, rendering the bicubic interpolation algorithm unable to accurately recover information at the individual pixel level. In contrast, the EPIM preserved sharp transitions at the mitochondrial edges, minimizing the blurring associated with image interpolation. The fluorescence lifetime distribution curves are depicted in Figure 3e. The bicubic method yielded smoother curves, mitigating the influence of the peaks present in the low-resolution images. The fluorescence lifetime histograms obtained using the EPIM were comparable to those from the bicubic method, also presenting a trend towards smoother curves. The spatially resolved analysis in Figure 3f–h also reflects the same trend.

Interestingly, the fluorescence lifetime represents the statistical outcome of the time taken by numerous molecules to return to their ground state, leading to slight variations in fluorescence intensity and lifetime among pixels representing the same component within a single measurement. Furthermore, minor fluctuations in fluorescence lifetime at the same pixel location can be observed across multiple measurements. Therefore, achieving absolute consistency in fluorescence intensity and lifetime for the same pixel across all measurements is impractical. However, the overall trend, specifically the fluorescence lifetime distribution histogram, remains largely unchanged during each measurement. Consequently, the fluorescence lifetime distribution histogram serves as a crucial criterion in the image reconstruction process.

**Figure 4:**
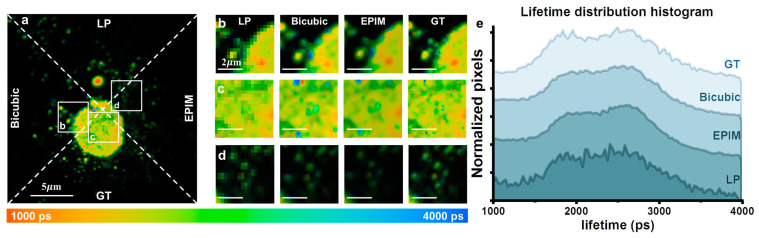
(**a**) Fluorescence lifetime image comparison of the LP (128 × 128), bicubic-interpolated, EPIM reconstruction, and GT images, divided by dotted lines. (**b**–**d**) ROIs of (**a**). (**e**) Fluorescence lifetime distribution histogram of (**a**).

We also conducted the imaging of nuclear pore complexes in fixed cellular samples to validate the effectiveness of the EPIM in other cellular structures. Low-resolution images were generated through downsampling to 128 × 128 pixels. The original 256 × 256 pixel images, followed by those obtained via bicubic interpolation, were utilized as GT images. The results presented in Figure 4 demonstrate that our method remains effective in this context.

### 3.3. Live-Cell Samples

**Figure 5:**
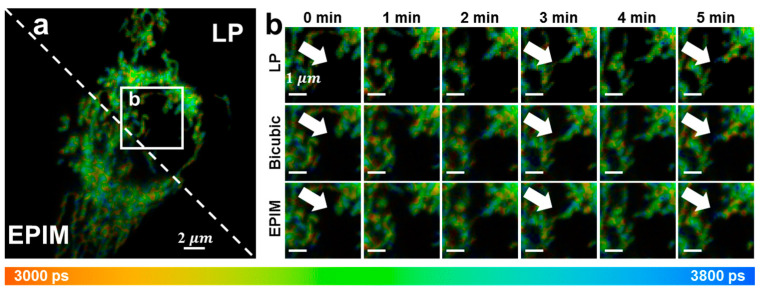
(**a**) Fluorescence lifetime image of live cells. The mitochondria are marked. (**b**) Regions of interest of (**a**). These images were collected continuously, with the first one starting at 0 min and the interval between each start time being 1 min. White arrows: rapid alterations in mitochondrial characteristics can be observed.

The imaging process for live-cell samples remained consistent with previous descriptions, with the exception of the use of a 633 nm laser as the excitation source and a 670/30 bandpass filter. To achieve the photon counts necessary for lifetime fitting, each image required 5–6 frames, with an accumulation time of less than 30 s. Consequently, an interval of 1 min was set between the initiation of each scan to enable the continuous imaging of the cells, with a total of six images collected. As depicted in Figure 5, we then presented the changes observed in the mitochondria within the cells. At 0–1 min, the mitochondria within the cells exhibited a linear distribution, with clear edges and high continuity. Over the subsequent minutes, mitochondrial motility and fragmentation (indicated by white arrows) were observed, occurring rapidly and resulting in subtle differences between each image. The interpolated images successfully revealed this process in high-pixel images, allowing the visualization of delicate subcellular structural changes. After continuous imaging over multiple scans, the cellular state deteriorated, with the mitochondria undergoing continuous fragmentation and swelling, ultimately leading to a large punctuate distribution with increasingly blurred boundaries. The acquired images directly demonstrated the changes in the mitochondria of live cells stimulated by laser exposure. The short acquisition times permitted dynamic and continuous observations.

## 4. Conclusions

Bicubic interpolation for processing low-resolution images often results in blurred edges, whereas image reconstruction algorithms based on EPIM are capable of retaining edge information and generating high-pixel images. Experimental results with fixed cellular samples validated the efficacy of this method in processing fluorescence lifetime images. To expedite the imaging process of lifetime images and capture subtle activities within live cells, we acquired low-pixel data and instead opted for an image reconstruction strategy to achieve high-pixel dynamic imaging. Despite remaining challenges, such as the inability to quantitatively assess the difference between the final interpolated image and the GT image, edge-preserving image reconstruction remains a rapid and convenient method for obtaining high-pixel lifetime images. This approach will likely find broader application in live-cell imaging in the future.

## Data Availability

The data that support the findings of this study are available from the corresponding authors upon reasonable request.

## Supplementary Materials

The following supporting information can be downloaded at https://www.mdpi.com/article/10.3390/bios15010043/s1.
