# Supplementary material for: Rapid Acquisition of High-Pixel Fluorescence Lifetime Images of Living Cells via Image Reconstruction Based on Edge-Preserving Interpolation
Source: Biosensors (Basel). 2025 Jan 13;15(1):43. doi: 10.3390/bios15010043 (PMC11763502; doi:10.3390/bios15010043)
Supplement: Supplementary file 1 [file biosensors-15-00043-s001.zip › biosensors-3364170-supplementary.pdf]

Rapid Acquisition of High-Pixel Fluorescence Lifetime Images of Living Cells via Image Reconstruction Based on Edge-Preserving Interpolation

Yinru Zhu [1], Yong Guo [1], Xinwei Gao [1], Qinglin Chen [1], Yingying Chen [1], Ruijie Xiang [1], Baichang Lin [1], Luwei Wang [1], Yuan Lu [2,*] and Wei Yan [1,*]

[1] State Key Laboratory of Radio Frequency Heterogeneous Integration (Shenzhen University), Key Laboratory of Optoelectronic Devices and Systems of Ministry of Education and Guangdong Province, College of Physics and Optoelectronic Engineering, Shenzhen University, Shenzhen 518060, China

[2] The 6th Affiliated Hospital of Shenzhen University, Huazhong University of Science and Technology Union Shenzhen Hospital, Shenzhen 518060, China

* Correspondence: chfsums@163.com (Y.L.); weiyan@szu.edu.cn (W.Y.)

Sample Preparation

Fluorescent Microspheres

The fluorescent microsphere sample was a mounted sample from the Thermo Fisher Scientific Company. We chose fluorescent microspheres with a size of 4.0 μm from position 1 of a TetraSpeck™ Fluorescent Microspheres Size Kit for imaging.

Fixed-Cell Sample

The fixed-cell sample was purchased from the Standard Imaging Company. The methods for immunofluorescence staining were as follows:

The BSC-1 cells were cultured in Dulbecco's Modified Eagle's Medium (DMEM) (Invitrogen, #11965-118) supplemented with 10% fetal bovine serum (FBS) (Gibco, #16010-159). To prevent bacterial contamination, 100 μg/ml penicillin and streptomycin (Invitrogen, #15140122) was added in the DMEM. The cells were grown under standard cell culture conditions (5% CO2, humidified atmosphere at 37°C). BSC-1 cells were plated on a #1.5 glass-bottom dish for 48 h before sample preparation. For cell passage, the cells were washed with pre-warmed PBS (Life Technologies, #14190500BT) 3 times and digested with 25% trypsin (Gibco, #25200-056) for 30 s. The BSC-1 l cell line was tested for potential mycoplasma contamination (MycoAlert, Lonza), and all tests showed negative results.

The cells were grown on 35 mm, #1.5 glass coverslips (SunBloss™, STGBD-035-1). To increase cell adhesion, we pre-treated glass-bottom dishes with fibronectin (SunBloss™, HXAR-01) for 1 h at 37°c. On the day of sample preparation, the cell density needed to be about 50%-70%. The cells were fixed with 37'C pre-warmed fixation buffer for 10 min, containing 4% paraformaldehyde and 0.1% glutaraldehyde (SunBloss™, HXKx01) in PBS. Then, the samples were washed three times with PBS. For quenching the background fluorescence, we incubated the cells with 2 ml 0.1% NaBH4 solution (SunBloss™, HXIK023) in PBS for 7 min. Optionally, they could be shaken on the shaker (<1Hz). The samples were washed three times with 2 ml PBS and then incubated for 30 min in PBS containing 5%

BSA and 0.5% Triton X-100 (SunBloss", HXKx02) at 37℃. All antibodies were diluted in the 5% BSA+0.5% triton solution. Next, we incubated the samples for 40 min with the appropriate dilution of primary antibodies—beta-tubulin (DSHB-E7)、Tom20 (abclonal, A19403), and LAMP1(abclonal, A22482)—at 25℃. After incubation with the primary antibodies, the cells were washed for 5 min with 2 ml PBS, three times. The samples were incubated for 60 min with the appropriate dilutions of secondary antibodies—abberior STAR RED, goat anti-mouse IgG (Abberior, STRED-1001-500UG), abberior STAR RED, and goat anti-rabbit IgG (STRED-1002-500UG)—at 25° c; the samples were protected from light. After being washed 3 times with PBS, the cells were fixed with post-fixation buffer for 10 min.

Living-Cell Samples

HeLa cells were cultured in DMEM containing 10% fetal bovine serum (FBS) and 1% penicillin/streptomycin; they were then stained with 10μl 75nM Lyso-Tracker Red dye. Following incubation for 10 minutes at 37°C in a 5% CO2 environment, the stained cells were washed three times with PBS buffer, replenished with DMEM, and further incubated for 10 minutes to maintain cell viability and reduce stress responses.

System Setup

The fluorescence lifetime imaging microscope (DCS-120, Becker & Hickl, GmbH, Germany) utilized in the study was a commercial laser scanning confocal microscope incorporating a time-correlated single-photon counting (TCSPC) module (SPC150, Becker & Hickl GmbH, Germany).

A mode-locked supercontinuum laser (SC-PRO-M, Wuhan Yangtze Soton Laser Co., Ltd, China) was utilized as the excitation source, featuring a pulse width of 6 picoseconds and a repetition rate of 80 MHz. This laser was coupled with an acousto-optic tunable filter (AOTF) to facilitate the selection of specific excitation wavelengths, enabling versatile applications across various samples.

For the fluorescent microspheres, fixed-cell samples, tissue slices, and living-cell samples, tailored excitation wavelengths of 570 nm, 633 nm, and 633 nm, respectively, were employed. To effectively isolate the signal from the reflected laser, appropriate bandpass or long-pass filters (620/60, 670/30, and 670/30, respectively) were implemented.

The optical resolution was maximized through the use of a high-performance 100× oil immersion objective lens (NA 1.40, Nikon, Japan). The resulting fluorescence signals were captured by a sensitive hybrid detector (HPM-100, Becker & Hickl GmbH, Germany), ensuring accurate and reliable measurements.

The entire fluorescence lifetime imaging process was seamlessly orchestrated by the SPCM64 software (version 9.76, Becker & Hickl, GmbH, Germany), providing intuitive control over data acquisition and processing.
